# Extracellular vesicles of *Emergomyces africanus* modulate host immune responses and reflect metabolic adaptations to nutrient availability

**DOI:** 10.1128/iai.00632-25

**Published:** 2026-02-17

**Authors:** Leandro Honorato, Albaniza Liuane Ribeiro do Nascimento Sabino, Jhon Jhamilton Artunduaga Bonilla, Susana Ruiz Mendoza, Julio Kornetz, Flavia C. G. dos Reis, Elaine R. Albergoni, Vinicius Alves, Susana Frases, Allan Jefferson Guimarães, Daniel Zamith-Miranda, Simone Sidoli, Joshua D. Nosanchuk, Marcio L. Rodrigues, Leonardo Nimrichter

**Affiliations:** 1 Departamento de Microbiologia Geral, Instituto de Microbiologia Paulo de Góes, Universidade Federal do Rio de Janeiro 28125, Rio de Janeiro, Brazil; 2 Department of Microbiology and Parasitology, Laboratory of Biochemistry and Immunology of Mycoses, Biomedical Institute, Fluminense Federal University 28110, Niterói, Rio de Janeiro, Brazil; 3 Instituto Carlos Chagas, Fundação Oswaldo Cruz (Fiocruz), Curitiba, Brazil; 4 Centro de Desenvolvimento Tecnológico em Saúde (CDTS), Fundação Oswaldo Cruz 37903, Rio de Janeiro, Brazil; 5 Laboratório de Biofísica de Fungos, Instituto de Biofísica Carlos Chagas Filho, Universidade Federal do Rio de Janeiro 28125, Rio de Janeiro, Brazil; 6 National Institute of Science and Technology in Human Pathogenic Fungi, São Paulo, Brazil; 7 Departments of Medicine and Microbiology and Immunology, Albert Einstein College of Medicine 2006, Bronx, New York, USA; 8 Department of Biochemistry, Albert Einstein College of Medicine 2006, Bronx, New York, USA; 9 Rede Micologia RJ, FAPERJ, Rio de Janeiro, Brazil; University of California Davis, Davis, California, USA

**Keywords:** *Emergomyces africanus*, extracellular vesicles, host immune responses, metabolic adaptations

## Abstract

*Emergomyces africanus* is a thermal dimorphic fungus and a leading cause of emergomycosis, a neglected infection primarily affecting immunocompromised individuals. Despite its clinical relevance, little is known about how *E. africanus* adapts to the host environment. Recent studies suggest that fungal extracellular vesicles (EVs) may contribute to host adaptation by modulating immune responses and transporting virulence factors. Here, we report the production and characterization of *E. africanus* EVs obtained under nutrient-rich and nutrient-limited media, mimicking environmental and host-like conditions. We also evaluated the effect of *E. africanus* EVs released in nutrient-limited media on bone marrow-derived dendritic cells (BMDCs) and bone marrow-derived macrophages (BMDMs). Under nutrient limitation, *E. africanus* released EVs enriched in virulence-associated proteins, including catalase, HSP60, and chitinase, whereas EVs from rich media carried proteins linked to anabolic pathways. Chitin-like structures and β-1,3-glucans were also detected in EVs released in nutrient-limited conditions. EVs from nutrient-limited conditions activated BMDCs, increased MHC-II and CD40 expression, and promoted a pro-inflammatory cytokine profile (IL-6 and TNF-α). In contrast, BMDMs exhibited elevated IL-10 levels, suggesting an anti-inflammatory phenotype. Remarkably, EV pre-treatment enhanced BMDM antifungal activity, significantly reducing *E. africanus* viability post-infection. These findings show that *E. africanus* dynamically adjusts its EV cargo in response to environmental cues, directly influencing immune modulation and fungal survival. Indeed, pre-treatment of the insect *Galleria mellonella* with EVs induced a protective response against a lethal inoculum of *Histoplasma capsulatum*. This work provides new insights into fungal adaptation and highlights EVs as potential therapeutic and vaccine platforms.

## INTRODUCTION

*Emergomyces* is a genus within the *Ajellomycetaceae* family that includes “Emmonsia-like” fungi responsible for causing emergomycosis (1). Species of this genus are soil-dwelling and have been identified across various continents, including Africa, North America, Europe, and Asia (1). *Emergomyces africanus* is endemic to Southern Africa, and human infections are linked with the high prevalence of immunocompromised individuals and challenging socio-economic conditions (1). In immunocompromised patients, this thermal dimorphic fungus causes skin lesions that typically appear as papules, plaques, nodules, or ulcers with widespread distribution. Although pulmonary infection is also common, the fungus rarely remains confined to the lungs, rapidly disseminating to other organs and leading to fatal systemic disease (2). As a neglected infection affecting vulnerable populations, emergomycosis represents a serious public health concern with partially unknown consequences (3). Therefore, understanding *Emergomyces* biology is necessary for developing new diagnostic and therapeutic strategies to address this health threat.

Extracellular vesicles (EVs) are lipid bilayer compartments secreted by both prokaryotic and eukaryotic cells (4). A number of studies revealed that EVs from diverse fungal species transport a wide variety of bioactive molecules, including proteins, RNAs, lipids, metabolites, glycans, and pigments (5–8). Their known roles in host cell activation, intercellular communication, and pathogenesis are closely linked to the diversity of their molecular composition, suggesting that other biological functions may also be associated with EV cargo variability (9, 10).

It is hypothesized that EVs dynamically adjust their cargo in response to external stimuli, reflecting metabolic and physiological adaptations in fungal cells. This concept is directly supported by Cleare and colleagues, demonstrating that *Histoplasma capsulatum* cultivation in distinct media leads to EVs with significantly different molecular profiles (11). Complementing these findings, Marina and colleagues revealed that EVs produced by *C. neoforman*s under nutrient-poor conditions exhibited larger hydrodynamic sizes, contained higher levels of virulence-associated compounds, and triggered stronger inflammatory responses compared to EVs produced in nutrient-rich media (12).

We recently demonstrated that *E. africanus* exhibits significant changes in morphology and the distribution of cell wall components when cultivated in Brain Heart Infusion (BHI), a nutrient-rich medium, when compared to Ham’s F-12 Nutrient Mixture (HMM) medium, used to mimic nutrient-limited and physiological conditions of the host environment (13). In the current study, we characterized the protein content of EVs released by *E. africanus* under both culture conditions and correlated their composition with the fungus’ ability to adapt to the host. EVs produced in nutrient-limited host-mimicking conditions were specifically analyzed due to their higher immunogenic profile, suggesting they can be harnessed for eliciting robust immune responses. Our findings bring new perspectives on how EVs may influence fungal adaptation and survival across different environments and open new possibilities for targeting fungal infections.

## RESULTS

### Characterization of EVs from *E. africanus*

**Fig 1:**
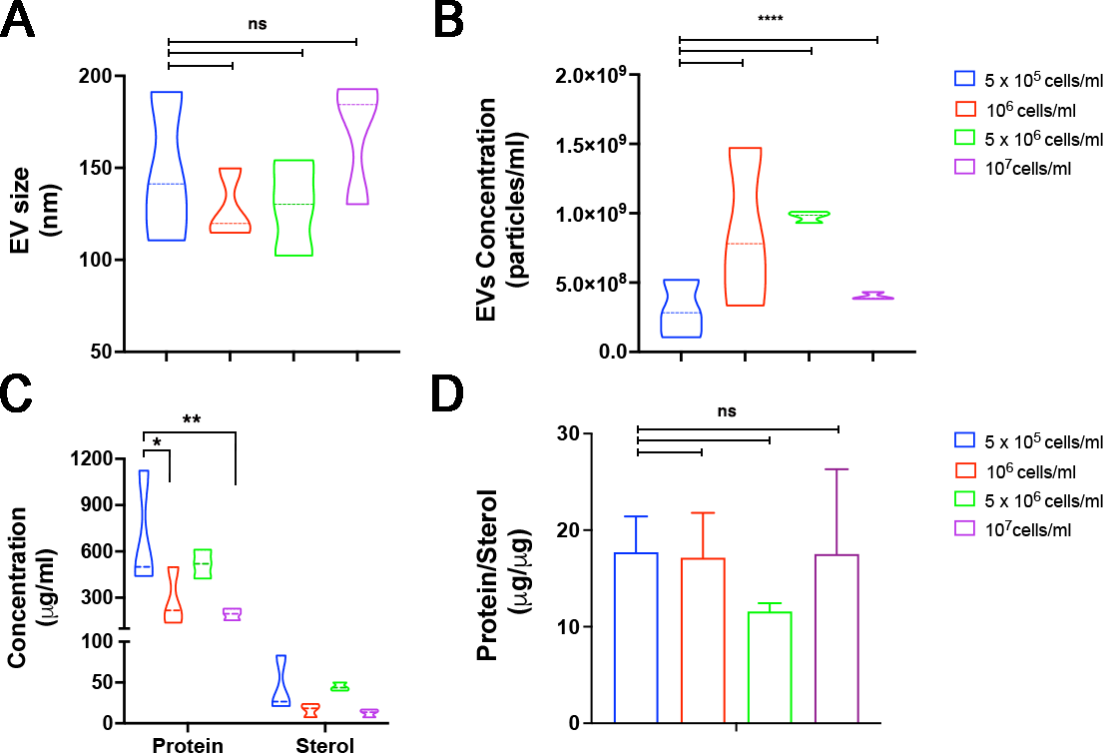
Characterization of EVs from *E. africanus* after cultivation (HMM medium) with different inocula. Nanoparticle tracking analysis (NTA) determinations of (**A**) size distribution and (**B**) particle concentration of EVs isolated from cultures in HMM initiated with different inocula. (**C**) Protein and sterol concentrations in EVs. (**D**) Protein/sterol ratio of EVs across different inoculum conditions. Results represent the average of three independent EV isolation experiments, and error bars represent the standard deviation (SD). Differences were considered significant using two-way analysis of variance (ANOVA) with *P* < 0.0327 (*), 0.0071 (**), and <0.0001(****) . Comparisons with no statistical difference were labeled as not significant (ns).

For these initial experiments, Ham’s F-12 Nutrient Mixture (HMM) medium was chosen because the strain used in this study exhibited faster growth in HMM when compared to BHI (13). To establish the optimal conditions for EV isolation, we initiated cultures of *E. africanus* with varying inoculum sizes (5 × 10^5^, 10^6^, 5 × 10^6^ or 10^7^ cells/mL of HMM). Our results showed that the EV diameter distribution among the distinct used inocula was similar (Fig. 1A), and the highest yield of EVs was achieved with an initial inoculum size of 1 × 10^6^ and 5 × 10^6^ cells/mL (Fig. 1B), when compared to the other inocula size tested. However, the variability in the number of particles was significantly smaller when 5 × 10^6^ and 1 × 10^7^ cells were used as the inocula size (Fig. 1B). Variation in the diameter of the EVs was also smaller for the initial inocula of 1 × 10^6^ and 5 × 10^6^ cells/mL, with average diameters of 128.1 and 128.8 nm, respectively. Protein and sterol concentrations of EVs were consistent across different batches for all inoculum conditions (Fig. 1C), except for the lowest density (5 × 10^5^ cells). Furthermore, the protein/sterol ratio remained similar within the different culture conditions (Fig. 1D). Considering primarily the smaller variability in diameter size, EVs concentration, and the protein/sterol ratio, the optimal inoculum condition was defined as 5 × 10⁶ cells/mL. EVs were then isolated from both HMM and BHI under the same conditions.

### Morphological and ultrastructural aspects of *E. africanus* and *E. africanus* EVs

**Fig 2:**
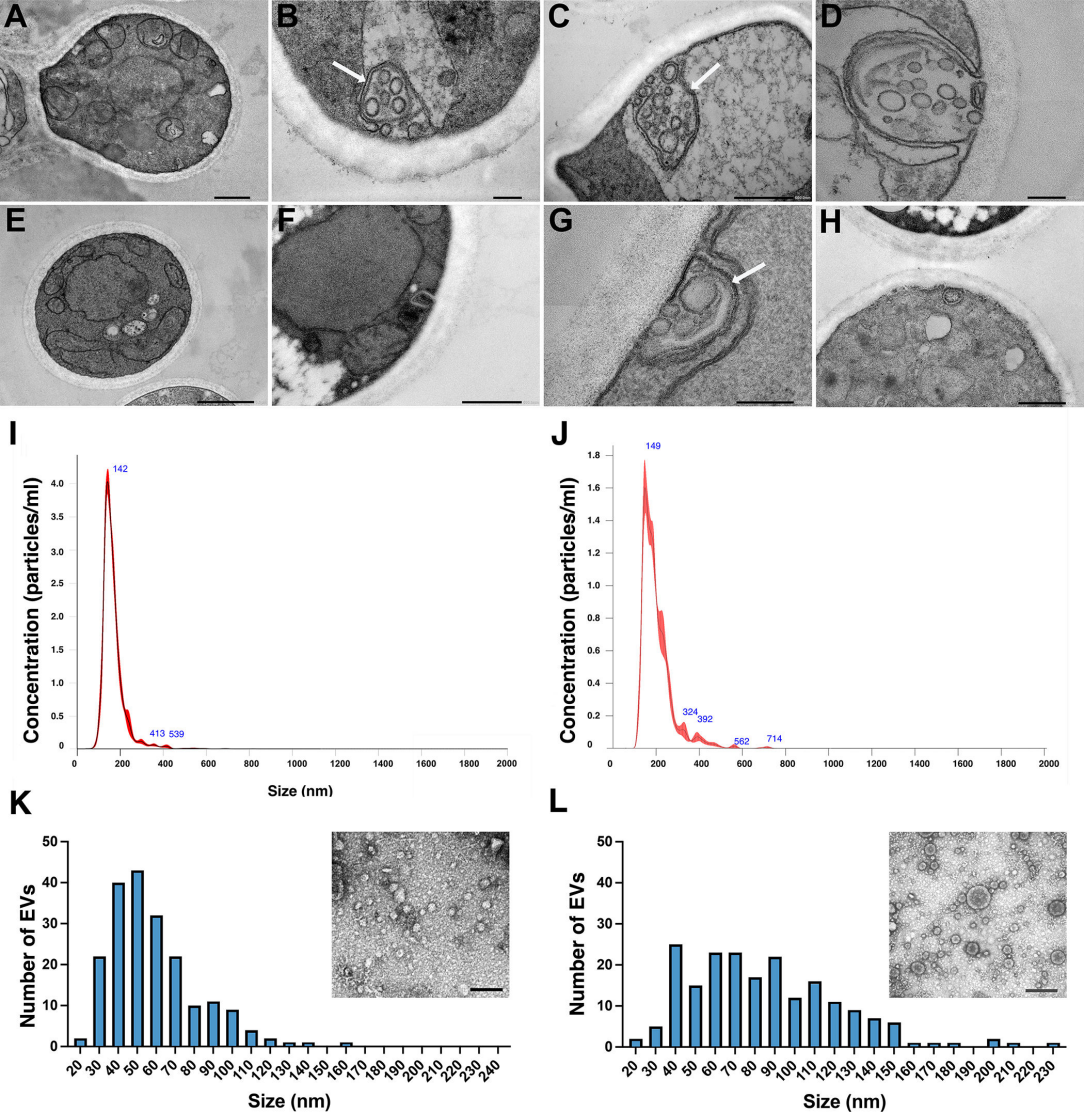
Morphological and ultrastructural analysis of *E. africanus* and its EVs. (**A–H**) TEM images showing MVB-like structures (MVBs-like; white arrows) and intraluminal vesicles in *E. africanus* cells grown in BHI (**A–D**) and HMM (**E–H**) media. (**I, J**) NTA of EVs isolated from BHI (**I**) and HMM (**J**) cultures, showing size distribution and particle concentration. (**K, L**) Histogram analyses and TEM images of isolated EVs obtained from BHI and HMM, respectively, display typical round and cup-shaped morphologies. Scale bars: 200 nm.

Transmission electron microscopy (TEM) images of *E. africanus* indicated that cultivating the fungus in BHI promotes a substantial increase in the formation of multivesicular body (MVB)-like structures, with a large number of intraluminal vesicles (Fig. 2A through D) when compared with HMM, where few MVBs and intraluminal vesicles were found (Fig. 2E through H). The MVB-like structures in *E. africanus* appeared to be in various stages of fusion with the plasma membrane, consistent with the formation of exosomes (8) in *E. africanus*, which was influenced by growth conditions. Although the size ranges of EVs obtained from cultures in different media were similar, their size distribution according to NTA analyses showed slight differences, corroborating this hypothesis. EVs derived from HMM medium exhibited a higher proportion of particles larger than 200 nm when compared to those released in BHI medium (Fig. 2I and J). The NTA data also showed a higher number of EVs were produced by the fungus in BHI (3.33 × 10^9^ ± 1.18 × 10^8^) compared to HMM (1.7 × 10^9^ ± 2.8 × 10^7^), consistent with the increased number of MVBs. Since EVs with sizes smaller than 50 nm are sometimes not accurately detected by the NTA equipment used in these experiments (14), we also evaluated their sizes using TEM micrographs and ImageJ software. TEM images of EVs confirmed the presence of typical round and cup-shaped structures (Fig. 2K and L), as reported for fungal EVs in previous studies (15). Although a higher number of small EVs was observed by TEM, especially in BHI medium (Fig. 2K), the size distribution corroborated the NTA findings, confirming a broader range of vesicles in HMM-derived EVs (Fig. 2L).

### Proteomic analysis indicates distinct protein profiles for *E. africanus* EVs cultivated in BHI versus HMM

**Fig 3:**
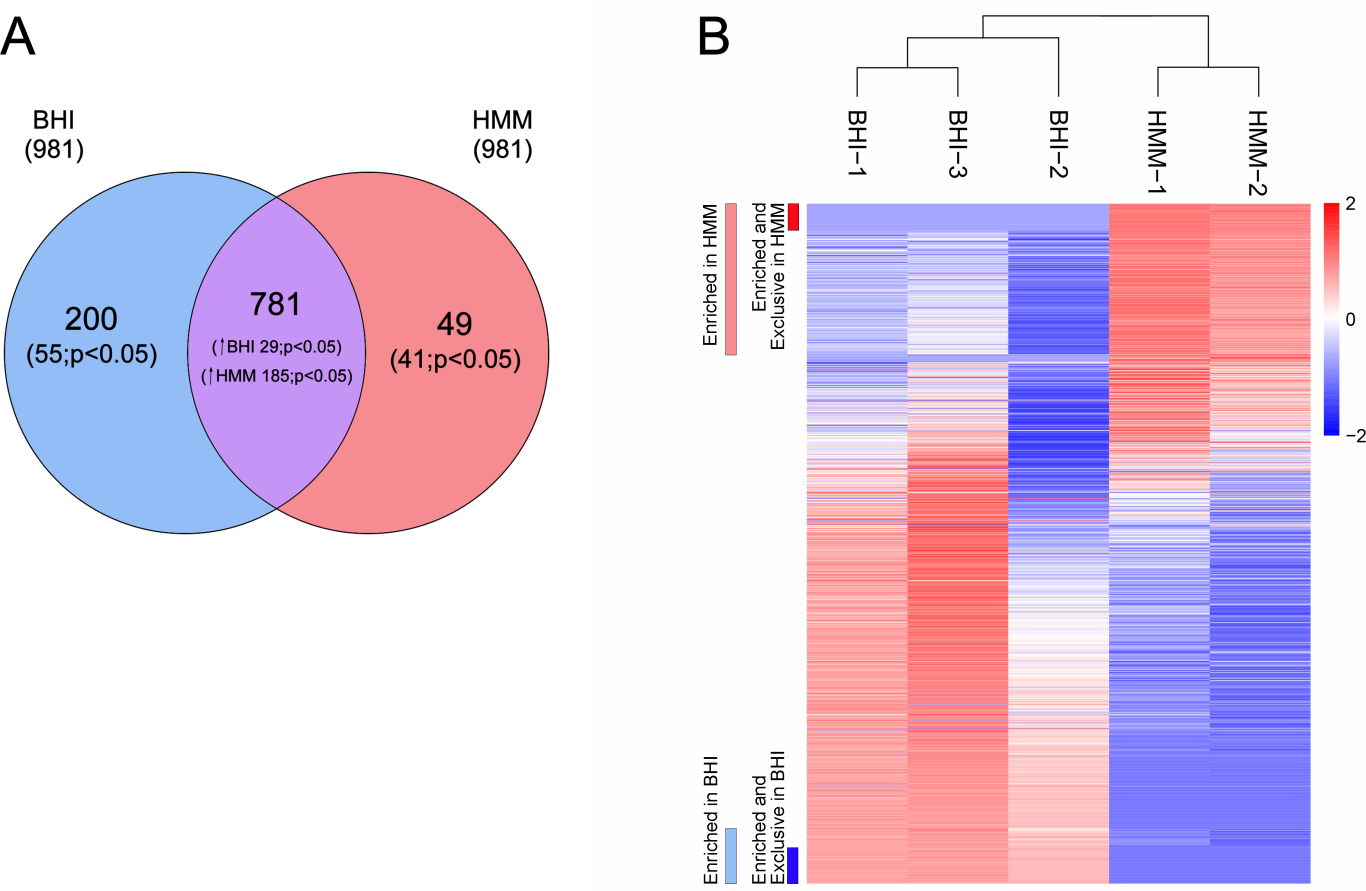
Proteomic profile of *E. africanus* EVs derived from BHI or HMM cultures. (**A**) Venn diagram showing the distribution of proteins identified in EVs derived from *E. africanus* cultured in BHI or HMM media. Of the total identified proteins, 781 were shared between both conditions, 200 were exclusive to BHI-derived EVs, and 49 were exclusive to HMM-derived EVs. (**B**) Heatmap depicting the relative abundance of proteins identified in EVs from both BHI and HMM media. Distinct clustering patterns highlight differences in protein expression based on the culture conditions.

**Fig 4:**
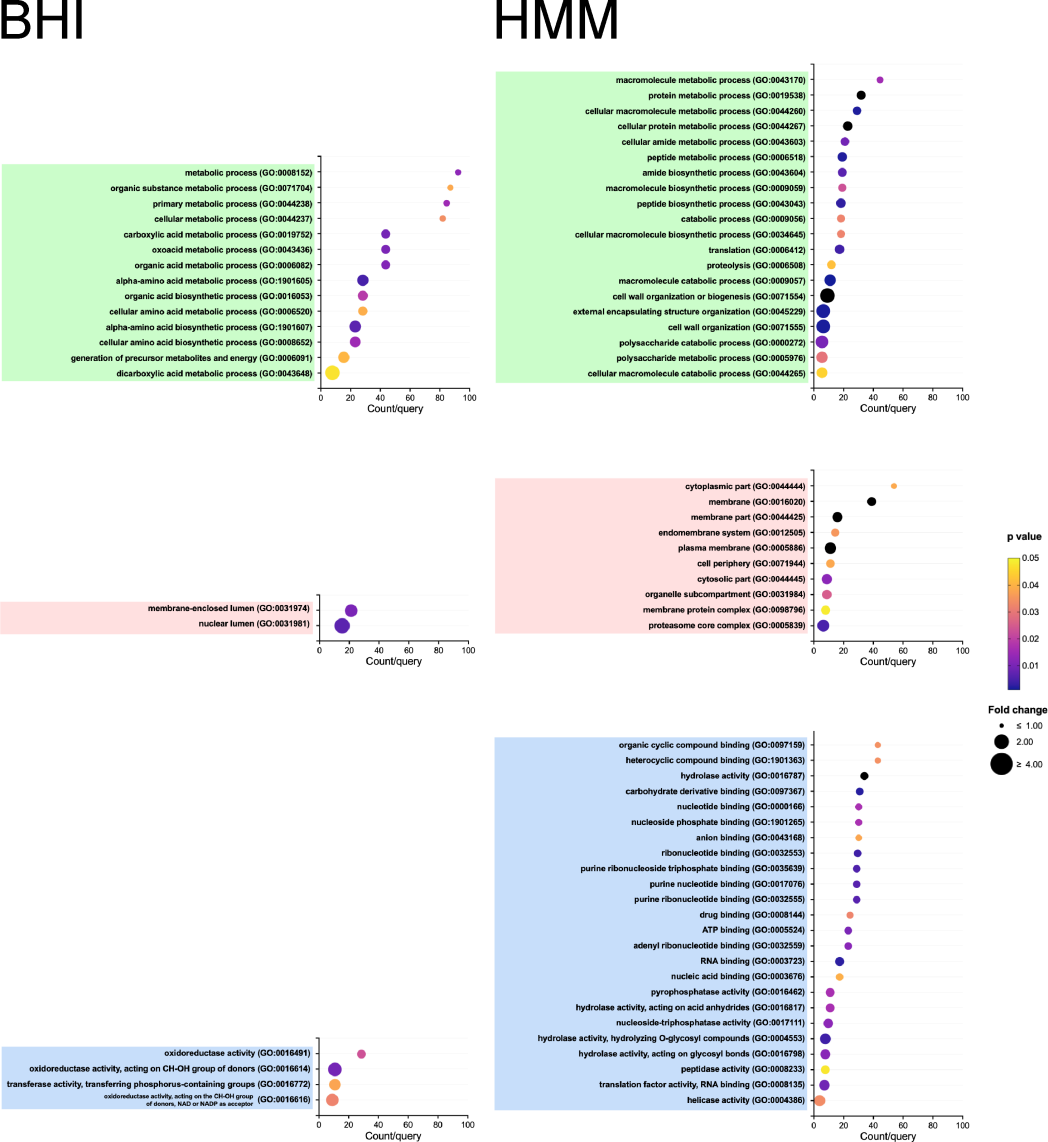
Proteomic analysis of *E. africanus* EVs derived from BHI or HMM cultures. Functional enrichment analysis of EV-associated proteins from each condition (BHI or HMM), demonstrating distinct biological processes (green), cellular components (pink), and molecular functions (blue).

A total of 1,030 distinct proteins were identified in the EVs produced by *E. africanus* under both culture conditions (Fig. 3A). From these, 781 proteins were shared by EVs from both, with 29 statistically enriched in EVs obtained from BHI culture, while 185 were enriched in EVs from the HMM medium. In the shared group, we detected several proteins involved with ER–Golgi–endosome–vacuole trafficking axis, including clathrin heavy chain, ENTH-, BAR-, and PH-domain-containing proteins, Vps1p, Vps10p, COPI and COPII members, Sec17p, along with partial t-SNARE and v-SNARE coiled-coil proteins. In addition, we found the vacuolar markers carboxypeptidase Y, dipeptidylaminopeptidase B, and Pep4-like proteases, as well as V-type proton ATPase subunits B and C. For the exclusive proteins, 200 proteins were found in EVs from BHI, with a total of 55 demonstrating statistical significance, while 49 proteins were exclusively found in EVs from HMM, with 41 being statistically significant (Fig. 3A). Figure 3B depicts a heat map showing all the analyzed proteins, with respective indications of exclusive and enriched or enriched proteins in EVs obtained from each condition. Remarkably, a very distinct profile was observed when the gene ontology (GO)/functions (biological process, BP, cellular component, CC, and molecular function, MF) of these enriched proteins under each culture condition (BHI and HMM) were compared. EVs isolated from *E. africanus* cultivated in BHI were enriched in macromolecule metabolic and catabolic process–related proteins, including amino acid, organic acid, and energy-related metabolism. The overrepresented GO terms included organic substance metabolic process, cellular amino acid metabolic process, generation of precursor metabolites and energy, and several categories related to carboxylic acid and oxoacid metabolism. In the cellular component category, enriched terms such as membrane-enclosed lumen and nuclear lumen indicate a predominance of proteins derived from intracellular organelles. The molecular function terms were enriched by oxidoreductase and transferase activities acting on CH–OH groups with NAD(P)+ as cofactors, consistent with active redox, central metabolic pathways, and biosynthetic specialization (Fig. 4). In contrast, EVs obtained from *E. africanus* grown in HMM were enriched in GO terms related to macromolecule metabolism and structural organization. The overrepresented biological processes included protein metabolic process, translation, proteolysis, and cell wall organization, as well as polysaccharide metabolic and catabolic processes. The cellular component terms, such as membrane, endomembrane system, proteasome core complex, and cell periphery, indicate that many of these proteins are associated with membrane-bound and endosomal origin structural compartments. From a molecular function perspective, HMM-derived EVs were enriched in hydrolase, peptidase, and helicase activities, along with proteins displaying ATP, RNA, and nucleotide-binding, and translation factor activity (Fig. 4). This profile suggests intense protein synthesis, processing, and turnover, for structural maintenance, as well as membrane remodeling and vesicle trafficking. Overall, BHI EVs emphasize cell-wall and protein turnover, whereas HMM EVs reflect metabolic and redox homeostasis, underscoring the condition-dependent EV biogenesis/composition and functional diversification. Furthermore, the presence of virulence-associated proteins in EVs derived from cultures in HMM, such as proteases, catalase, HSP60, and chitinase, leads us to hypothesize that these EVs could be more immunogenic compared to those released by *E. africanus* cultivated in BHI. Although the proteomic data rely on predicted annotations, the analyses provide a reliable basis for future experimental validation. All analyses derived from the proteomic data sets described here are available in Mendeley Data (16).

### β-1,3-glucan and chitin-like content in EVs released by *E. africanus* cultivated in HMM medium

To assess the presence of chitin oligomers and β-1,3-glucans, we used wheat germ agglutinin (WGA) and dectin-1-Fc(IgG) fusion proteins, respectively (17). These analyses confirmed the presence of both structures in *E. africanus* EVs (Table 1). Notably, dectin-1-Fc binding assays revealed that the β-1,3-glucan content was significantly lower than the amount of chitin-like structures, indicating that chitin is more abundant in these vesicles under the tested conditions.

**TABLE 1:** Chitin and β-1,3-glucan content in EVs^*a*^

Strain	Chitin-related structures (ng/µg of ptn)	β-glucan (ng/µg of ptn)
*E. africanus*	83.2 ± 9.8	51.5 ± 3.9

^*a*^ Average ± SD (*n* = 9) is given.

### Bioactivity of *E. africanus* EVs

**Fig 5:**
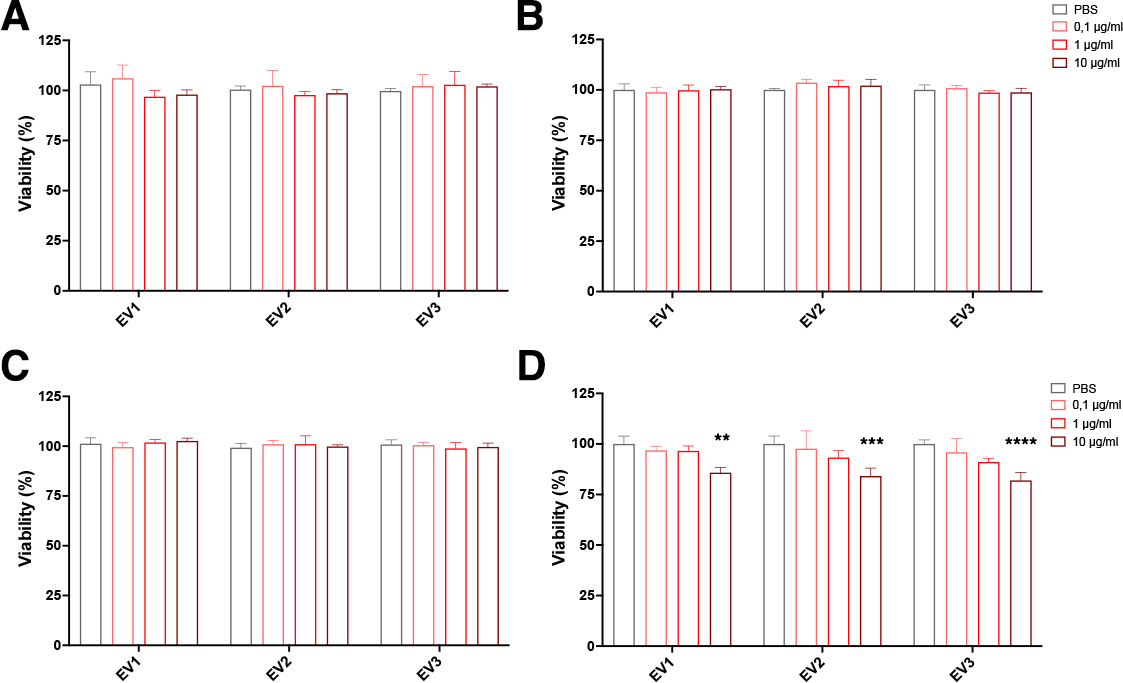
Cytotoxicity of *E. africanus* EVs. The phagocytes: BMDCs (**A**), BMDMs (**B**), AMJ2 (**C**), and the lung epithelial cell A549 (**D**) were incubated with different concentrations of EVs produced in HMM (0.1, 1, or 10 µg/mL) or vehicle (phosphate-buffered saline [PBS]) as a control for 24 h in a 96-well plate. Three independent EV preparations (EV1, EV2, and EV3) were tested. After that, cell viability was determined spectrophotometrically at 540 nm (ABS, absorbance) by MTT assay. Data shown are the mean ± SD of three independent experiments performed in triplicate. Differences were considered significant using two-way ANOVA with *P* < 0.0012 (**), 0.0004 (***), and 0.0001 (****) compared with the PBS group.

Since EVs produced by *E. africanus* cultivated in HMM medium carry several proteins associated with adaptation to host environmental conditions, including those associated with fungal virulence, we evaluated their toxicity and ability to modulate the function of phagocytes. Bone marrow-derived macrophages (BMDMs), bone marrow-derived dendritic cells (BMDCs), alveolar macrophages (AMJ2-C11), and lung epithelial cells (A549) were incubated with defined concentrations of *E. africanus* EVs. None of the EVs concentrations modified the metabolic activity of the phagocytes; therefore, cell viability was not impacted in these cells (Fig. 5A through C). However, the epithelial cell line exhibited a slight reduction in metabolic activity (16.1% ± 1.9%) when exposed to the highest EV concentration (10 μg/mL, based on protein content) (Fig. 5D). Given the low toxicity observed, a concentration of 10 μg/mL was selected for the experiments to determine the cytokine production.

### EVs released by *E. africanus* activate murine phagocytes

**Fig 6:**
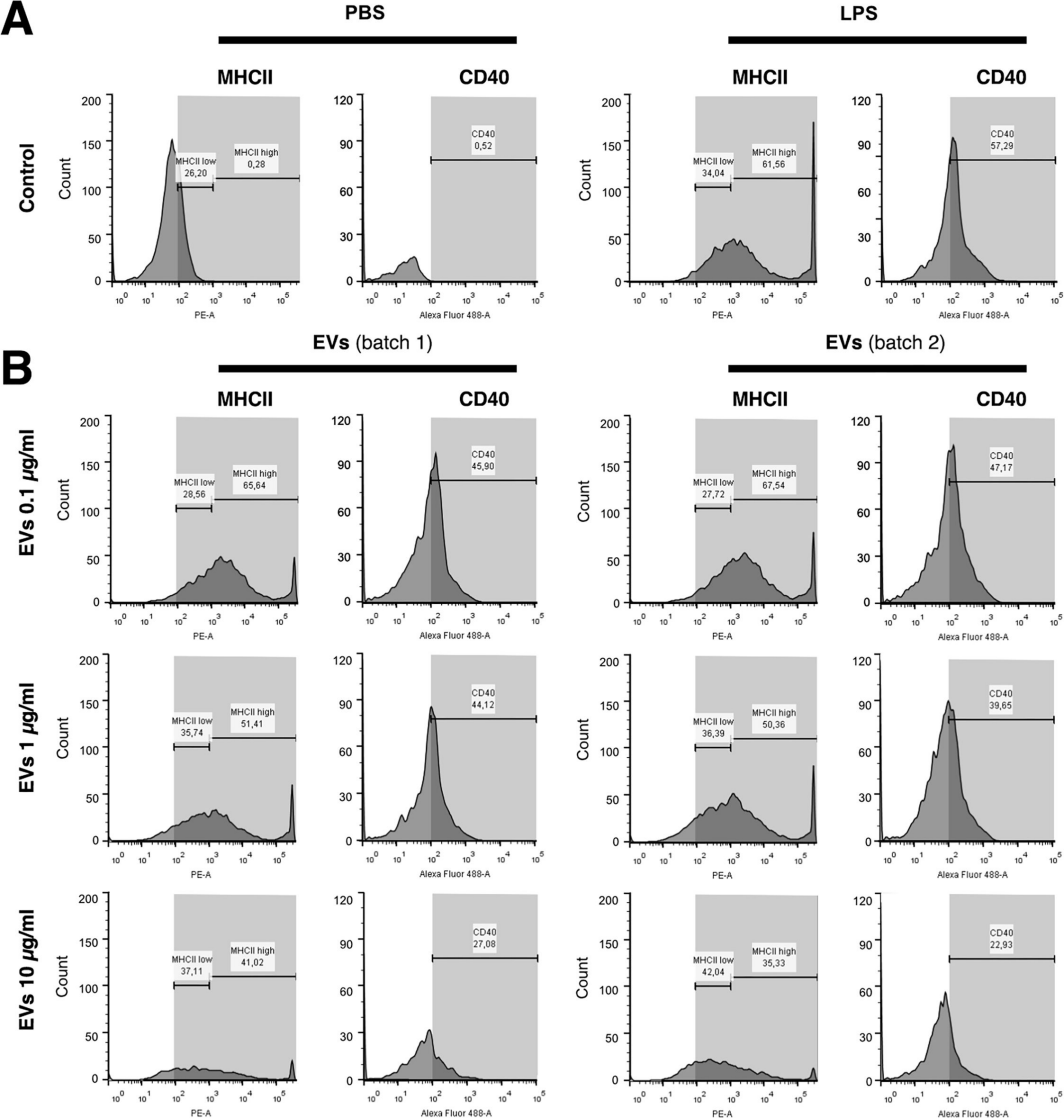
*E. africanus* EVs induce activation of murine BMDCs. BMDCs were incubated for 24 h with (**A**) PBS (negative control), or LPS (100 ng/mL, positive control) or (**B**) different concentrations of two batches of *E. africanus* EVs (0.1, 1, or 10 µg/mL). Flow cytometry analysis was performed to assess surface expression of MHC-II and the co-stimulatory molecule CD40. Histograms represent the expression levels of MHC-II and CD40 on BMDCs. EV-treated cells showed a dose-dependent increase in MHC-II^high^ and CD40 expression compared to the PBS control.

To investigate the effect of *E. africanus* EVs on BMDCs activation, we first evaluated the surface expression levels of MHC-II and the co-stimulatory molecule CD40. Figure 6 shows that BMDCs incubated with PBS (control) displayed less than 1% of MHC-II^high^ cells, as a baseline of non-activated cells (Fig. 6). Treatment with lipopolysaccharide (LPS) (positive control) significantly increased the MHC-II^high^ population (Fig. 6). Consistent with an activated profile, BMDCs treated with *E. africanus* EVs (10 µg/mL) displayed a significant increase in surface MHC-II^high^, which increased in a dose-response manner. Cells treated with the highest EV concentration exhibited values comparable to LPS-treated cells (~65% of cells MHC-II^high^). Likewise, EV-treated BMDCs showed a dose-dependent increase in CD40 expression, with values similar to those observed for LPS treatment (Fig. 6).

**Fig 7:**
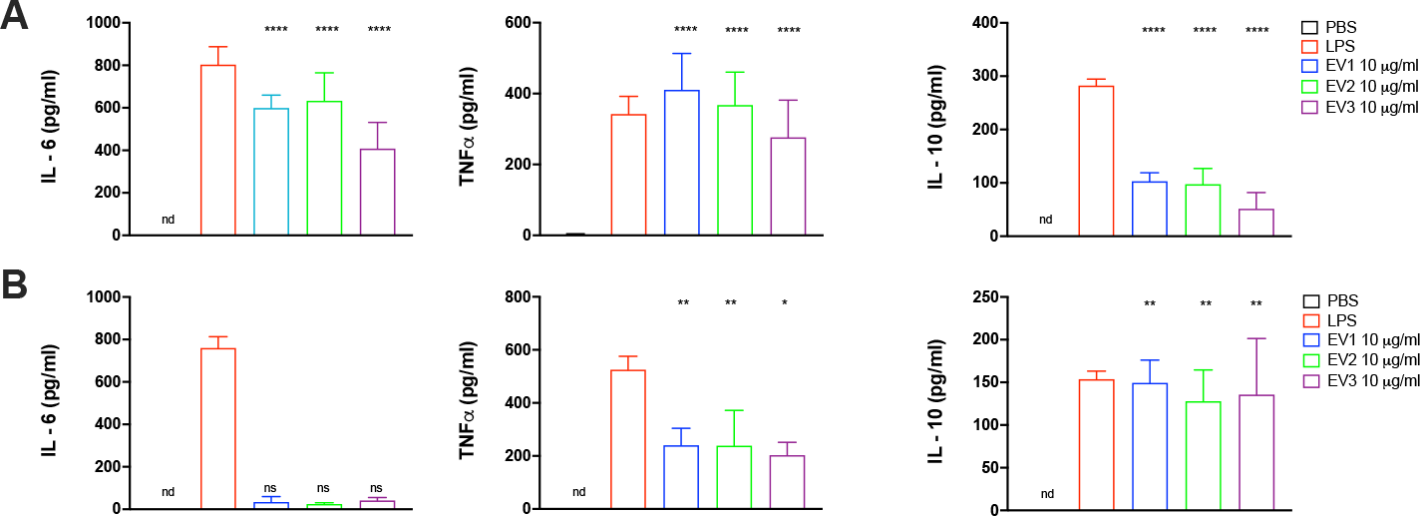
Differential cytokine production by BMDCs and BMDMs in response to *E. africanus* EVs. (**A**) BMDCs and (**B**) BMDMs were incubated for 24 h with *E. africanus* EVs (0.1, 1, or 10 µg/mL), PBS (negative control), or LPS (100 ng/mL, positive control). Cytokine levels of IL-6, TNF-α, and IL-10 in the culture supernatants were quantified by ELISA. Data shown are the average ± SD of two independent experiments. Differences were considered significant using ordinary one-way ANOVA with *P* = 0.0223 (*), 0.0086 (**), 0.0003 (***), and <0.0001 (****) compared with the PBS group (nd: non-detectable, ns: not significant).

The cytokine production by BMDCs and BMDMs treated with EVs from *E. africanus* was also investigated. BMDCs treated with *E. africanus* EVs (10 μg/mL, based on protein content) produced IL-6 and TNF-α at levels comparable to LPS treatment (Fig. 7A). However, IL-10 production was significantly lower than that observed with LPS, suggesting that the EVs promote a pro-inflammatory response in BMDCs (Fig. 7A). In contrast, BMDMs treated with *E. africanus* EVs produced very low levels of IL-6, with no statistical differences when compared to control cells (Fig. 7B). Additionally, TNF-α production in BMDMs was lower compared to both LPS-treated cells and EV-treated BMDCs. Remarkably, IL-10 was significantly elevated in BMDMs treated with the EVs, reaching levels compared to those induced by LPS treatment (Fig. 7B), indicating a less inflammatory profile of BMDMs when compared to BMDCs. These data indicate that *E. africanus* EVs differentially modulate BMDCs and BMDM activities.

### *E. africanus* EVs enhanced the ability of BMDM to kill yeast cells

**Fig 8:**
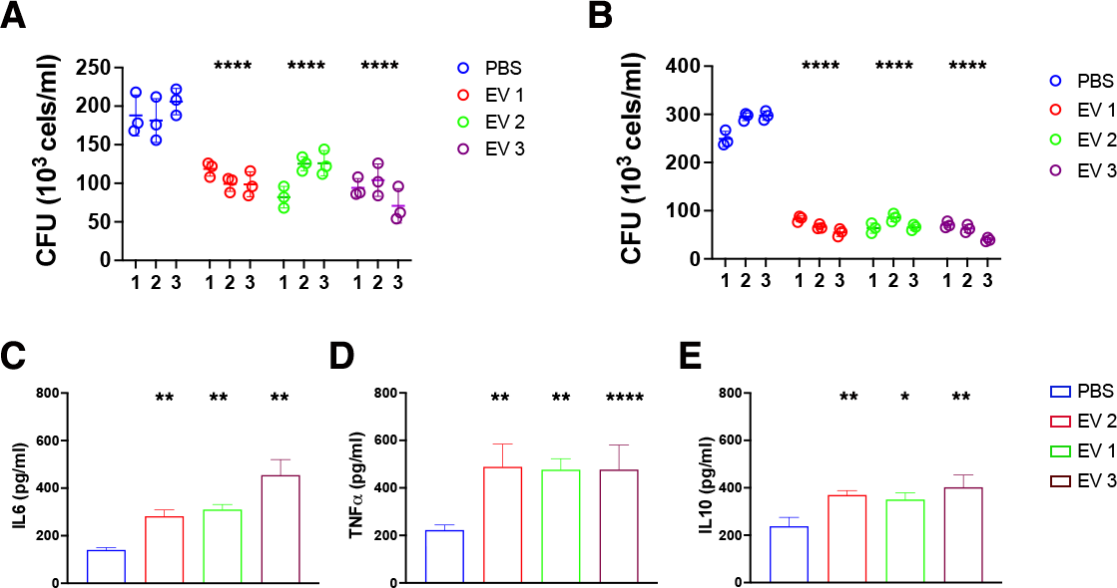
EVs from *E. africanus* enhance the fungicidal activity and the cytokine production of BMDMs. BMDM (10^6^ cells) were pre-treated for 24 h with *E. africanus* EVs (10 μg/mL) or PBS, washed, and subsequently infected with *E. africanus* yeasts at 1:1 yeast-to-macrophage ratio. After 1 day (**A**) or 4 days (**B**) post-infection (p.i.), cells were washed, lysed, and the number of viable intracellular yeasts was determined by CFU assay. The production of IL-6 (**C**), TNF-α (**D**), and IL-10 (**E**) was measured in the supernatants collected on day 4 post-infection by ELISA. Data represent mean ± SD from at least three independent experiments. Differences were considered significant using two-way ANOVA with *P* < 0.05 (*), 0.01 (**), and 0.001 (****).

Since macrophages are central players during antifungal immune responses and fungal elimination, we investigated the effect of *E. africanus* EVs on BMDM’s ability to control intracellular yeast infection. Pre-treatment with EVs significantly enhanced macrophage antifungal activity, resulting in a 47.8% ± 9.9% and 71% ± 3.9% reduction in CFU after 1 and 4 days of incubation, respectively (Fig. 8A and B). Additionally, EV exposure prior to *E. africanus* infection increased cytokine production of IL-6, TNF-α, and IL-10, indicating that the modulatory effect of EV-treated BMDMs differs when yeast is present (Fig. 8C through E).

### *E. africanus* EVs protect *Galleria mellonella* larvae from lethal infection with *Histoplasma capsulatum*

**Fig 9:**
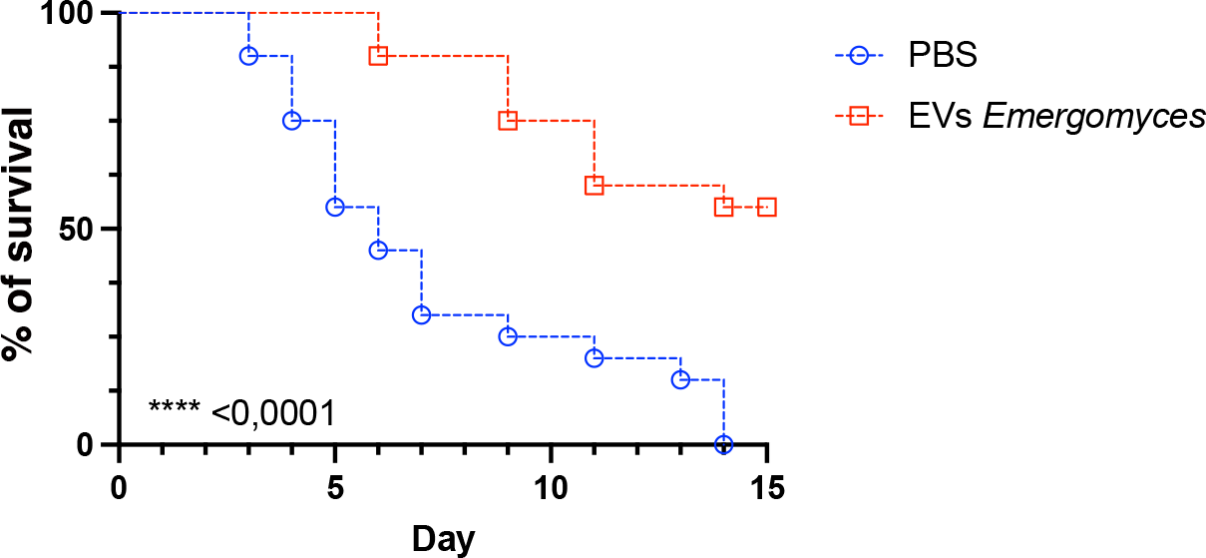
EVs from *E. africanus* protect *G. mellonella* larvae against lethal histoplasmosis. *G. mellonella* larvae (*n* = 20) were pretreated with *E. africanus* EVs (10 μL of a 100 µg/mL suspension, based on protein content) and then infected 2 days later with 5 × 10^6^ yeasts of *H. capsulatum* G217b (lethal inoculum). Statistical analysis was performed between groups using the log-rank (Mantel-Cox) test.

Since a high dose of *E. africanus* is not able to kill *Galleria mellonella* larvae (13) in our infection model, we used another dimorphic fungus from the Ajellomycetaceae family, *Histoplasma capsulatum*, as a surrogate to investigate the effect of *E. africanus* EVs on the course of a fungal infection. The survival curve (Fig. 9) demonstrates that larvae pre-treated with *E. africanus* EVs exhibited significantly increased survival compared to the PBS-treated group, in which the larvae succumbed to infection by day 14. EV-treated larvae showed a delayed mortality rate and enhanced overall survival (60%). These results indicate that *E. africanus* EVs induced a protective effect in *G. mellonella*, reducing the lethality of the *H. capsulatum* infection.

## DISCUSSION

In a previous study, we characterized ultrastructural differences that occurred when yeasts of *E. africanus* were cultivated under nutrient-rich conditions (BHI medium) compared to environments that mimic the physiological conditions encountered in the human lungs, such as the host-mimicking HMM medium (13). Here, we analyzed a higher number of cells and concluded that under nutrient-rich conditions, this organism increases the amount of MVB-like structures, which could be associated with distinct metabolic pathways. These results led us to investigate the potential involvement of EV biogenesis and cargo during *E. africanus* environmental adaptation. We first identified the inoculum conditions in the HHM medium that provided the highest yield of EVs with the least variability in number and size. Based on these results, we compared the size, morphology, and protein content of EVs released by *E. africanus* cultivated in BHI and HMM. Corroborating previous studies, *E. africanus* EVs displayed morphology and sizes compatible with EVs from other fungal species, with round compartments ranging from 40 to 400 nm (15). In addition, the higher number of small EVs released under rich-medium (BHI) was concordant with the increased presence of structures resembling MVBs, in contrast to the higher number of medium-sized EVs observed under nutrient-limited conditions (HMM). Together, these data supported the hypothesis of distinct cargo in EVs being produced under BHI versus HMM culture conditions.

Indeed, the comparative analysis of EV proteomics from *E. africanus* grown in HMM or BHI media highlights distinct metabolic strategies adopted by the fungus in response to nutrient availability. Although the predominance of proteolysis- and catabolism-related proteins in HMM-derived EVs suggests a metabolic state favoring resource conservation and adaptive remodeling, likely in response to nutrient limitations (18). In addition, the presence of proteins involved in cell wall biogenesis and membrane functions in these EVs may reflect structural modifications necessary for survival under limited conditions. Conversely, the enrichment of biosynthetic enzymes in BHI-derived EVs indicates an anabolic metabolic shift, where the fungus utilizes available nutrients to synthesize essential amino acids. These findings support the hypothesis that *E. africanus* modulates EV cargo composition in response to environmental conditions, indicating their involvement in fungal adaptation. Since the proteins characterized in EVs produced under nutrient restriction and host-mimicking conditions were associated with fungal adaptation to the host and included virulence factors, we investigated their impact on phagocytes and lung epithelial cells. As observed in previous studies with EVs produced by other fungal species (19–22), EVs from *E. africanus* did not cause any metabolic damage to phagocytes. However, a small but significant decrease in the metabolic activity of epithelial cells was observed at the highest EV concentration (10 µg/mL), indicating that these compartments could impact the interaction between *E. africanus* and the lung tissue.

Little is known about the mechanisms of fungal EV biogenesis. To explore this, we analyzed the proteins shared by EVs under the two nutritional conditions. Despite the TEM images showing MVB-like structures, no core ESCRT components were detected. However, we identified proteins classically associated with protein and lipid flux along the ER–Golgi–endosome–vacuole trafficking axis. For instance, canonical modules involved in membrane curvature and cargo selection, including clathrin heavy chain and ENTH-, BAR-, and PH-domain-containing proteins, were present. Their detection in EVs suggests that exported cargo may either transit endocytic checkpoints en route to MVBs or bud directly from the plasma membrane (23–25). The presence of the dynamin-like GTPase Vps1 further supports this model, as it could drive endomembrane scission and assist in intraluminal vesicle formation independently of ESCRT (26). Signatures of the secretory pathway were also evident. All five essential COPII subunits (Sar1, Sec23, Sec24, Sec13, and Sec31) were found. Proteins associated with the retrograde pathway were also identified, including COPI subunits α, γ, δ, and ε, suggesting active Golgi–ER recycling. We also detected Sec17p, a protein involved in SNARE complex disassembly, along with partial t-SNARE and v-SNARE coiled-coil proteins, implying the presence of fusion machinery components in fungal EVs (27). Lastly, we observed vacuolar markers and enzymes, reinforcing vacuolar involvement in EV biogenesis (18, 28). These include, for instance, carboxypeptidase Y, recognized by Vps10p for delivery to the vacuole or MVBs, and dipeptidylaminopeptidase B and Pep4-like proteases, both classical markers of the yeast vacuole (18, 26). Additionally, V-type proton ATPase subunits B and C were found under both nutritional conditions. These findings support a model in which the vacuole serves not only as a degradative endpoint but also as a hub intersecting with the EV export route, contributing to cargo selection and delivery. In addition, they underscore the mechanistic complexity of fungal EV biogenesis, suggesting a dynamic interplay between secretory, endocytic, and vacuolar pathways in shaping EV composition and function.

Fungal EVs are well-known modulators of innate immunity due to their cargo containing pathogen-associated molecular patterns and immunogenic molecules (17, 19, 21, 22, 29). Their role in activating BMDCs and BMDMs has been extensively reported (12, 19, 22, 30–32). Our results showed that treatment of BMDCs with *E. africanus* EVs resulted in activation of these cells, with increased expression of MHCII and the co-stimulatory molecule CD40, and release of pro-inflammatory cytokines. This response was similar to the effects of EVs from *Candida albicans*, *Candida auris*, and *Sporothrix brasiliensis*, but contrasted with the lack of activation observed by *Paracoccidioides brasiliensis* EVs (22, 30, 33, 34). In macrophages, however, *E. africanus* EVs triggered a distinct response characterized by a reduced or absent IL-6 production and elevated IL-10 levels. This pattern indicated a shift toward an anti-inflammatory profile, which may favor fungal survival within these cells. This resembles the immunosuppressive response triggered by *H. capsulatum* EVs in macrophages (35), suggesting that *E. africanus* may adopt immune evasion strategies similar to those observed during histoplasmosis. Conversely, this contrasts with the pro-inflammatory responses typically induced by EVs from other fungal pathogens, including *C. neoformans* and *C. albicans* (19, 22). We recently demonstrated that EVs from *C. albicans* carry chitin oligomers and β-1,3-glucan (17). The polysaccharide β-1,3-glucan activates dectin-1, promoting a pro-inflammatory response (36). However, IL-10 production is known to be stimulated by chitin, another structural cell wall polysaccharide present in fungal organisms (37). We also showed that EVs from mutants of *C. albicans* expressing higher amounts of chitin oligomers are potent inducers of IL-10 in BMDCs (17). Consistent with this, EVs released by *E. africanus* contain higher levels of chitin oligomers and lower amounts of β-1,3-glucan when compared to wild-type *C. albicans* EVs, which may explain the cytokine profile observed specifically in macrophages. These contrasting cytokine responses suggest that BMDCs and BMDMs are differentially affected by *E. africanus* EVs, possibly due to distinct receptor engagement and signaling pathways, resulting in varied immune outcomes. Despite the observed anti-inflammatory profile, pre-treatment of macrophages with these EVs was associated with a reduction of *E. africanus* viability. The number of CFUs was even lower after 4 days of infection compared to 24 h, whereas the fungal burden in untreated macrophages increased from the first to the fourth day post-infection. These data indicated that despite the anti-inflammatory profile induced by these EVs, they still enhanced the ability of phagocytes to combat fungal growth. We cannot rule out that the interaction of *E. africanus* and the macrophage may promote its activation, likely due to the exposure of different ligands than the EVs. In fact, a distinct cytokine profile, including an increase of IL-6 and TNFα, was observed 4 days after infection with *E. africanus* as the pre-treatment with EVs promoted an additive effect on the production of the three cytokines, when compared to untreated infected macrophages and corroborating with the antifungal activity of these cells. Previous studies have demonstrated that pre-treatment of insects and mice with *C. albicans* EVs resulted in a protective effect against lethal candidiasis (17, 21). A protective effect in *G. mellonella* was also observed for EVs released by *C. neoformans* and *Aspergillus flavus* (20, 31, 38). Since the *E. africanus* strain used in our study is unable to establish a lethal infection in *G. mellonella* larvae (13), we evaluated the protective effect of *E. africanus* EVs by pre-treating larvae and subsequently challenging them with a lethal inoculum of *H. capsulatum*. The protective effect observed in this model suggests that *E. africanus* EVs could potentially be explored as components in vaccine formulations aimed at preventing other fungal diseases, such as histoplasmosis.

Our study reports the first isolation of *E. africanus* EVs and correlates their composition with significant ultrastructural changes and the fungus’s ability to adapt to altered nutritional conditions. The higher content of MVBs indicates that a number of proteins are targeted to vacuolar compartments, aligning with the enrichment of ubiquitin and polyubiquitin proteins in HMM culture. The biogenesis of fungal EVs is still poorly understood, but our morphological data suggest that the *E. africanus* EVs may correspond mostly to exosomes, as concluded by the observation of an apparent fusion between MVBs and the plasma membrane and consequent release of vesicles into the periplasm. In addition, the activation of phagocytes by these EVs supports the idea that these compartments could be exploited in vaccine formulations against emergomycosis and maybe other fungal diseases. Specifically, the role of IL-10 in vaccine-related immunity efficacy must be carefully considered, as it plays a crucial role in modulating immune responses and preventing excessive inflammation. However, depending on the complexity of the immune response, an overly polarized TH1 response may create a hyperinflammatory microenvironment, potentially causing tissue damage and compromising the host. Conversely, a vaccine that incorporates epitopes capable of stimulating both TH1 and TH2 responses could achieve a balance between protective immunity and controlled inflammation, as previously demonstrated for *H. capsulatum* in mice immunized with HSP60 (39).

## MATERIALS AND METHODS

### Fungal strain and growth conditions

The clinical isolate of *E. africanus* was kindly provided by Dr. Nancy Wengenack (Microbiology and Laboratory Medicine and Pathology, Mayo Clinic, USA), and the *H. capsulatum* strain G-217B was obtained from the ATCC (ATCC 26032). The strains were stored in BHI blood agar (Sigma, USA) at −80°C. For fungal growth, an aliquot was thawed and cultured in BHI or HMM (Ham’s F-12 Nutrient Mixture (Gibco, US) supplemented with glucose (Sigma, US) 1.82g/L, glutamic acid (Vetec, Brazil) 1 g/L, HEPES (Gibco, US) 6.5 g/L, and L-Cysteine (Sigma, US) 8.4 mg/L) (40) at 37°C with agitation for 4 days (180 rpm). For the experiments, *E. africanus* was cultured in 1 L of HMM at an initial cellular concentration of 5 × 10^6^ cells/mL for 4 days at 37°C with agitation for 4 days (180 rpm). All experiments were performed using the yeast form of the fungus.

### Preparation of fungal EVs

EV isolation was performed following protocols established in our laboratory (41). Initially, optimal culture conditions for EV production were determined using HMM. Cell suspensions with increasing yeast inocula (5 × 10⁵, 10⁶, 5 × 10⁶, or 10⁷) were transferred into 1,000 mL of HMM and incubated for 4 days as described above. The culture supernatant was subsequently collected and processed for EV isolation. Briefly, cultures were centrifuged at 5,000 × *g* for 15 min at 4°C in a Beckman Avanti J-E Centrifuge. The supernatants were collected and further centrifuged at 15,000 × *g* for 15 min at 4°C in a Beckman Avanti J-E Centrifuge to remove cell debris. Residual cells and debris were removed after a step of filtration using a 0.45 μm membrane filter (Merck Millipore, US). The cell-free supernatant was concentrated about 50 times using a VivaFlow system (100 kDa membrane, Sartorius, US). The concentrated supernatant (20 mL) was then ultracentrifuged at 100,000 × *g* for 1 h at 4°C in a Beckman Optima LE-80K ultracentrifuge with a 70 ti rotor. The resultant pellet was washed twice with PBS, pH 7.4, at 100,000 × *g* for 1 h at 4°C. The collected fungal EVs were suspended in PBS, and aliquots were plated onto BHI agar (Sigma, US) and incubated at 37°C for 15 days to confirm the absence of contaminants, verified by the absence of colony growth (sterility of the sample). Quantification of EVs was carried out using the quantitative Amplex Red Sterol Assay Kit (Invitrogen, US) and the bicinchoninic acid (BCA) Protein Assay Kit (ThermoFisher, US) following the manufacturer’s instructions. EVs were stored at –80°C, and all experiments were performed within 2 weeks.

### Nanoparticle tracking analysis

EVs concentration and size distribution were measured using ZetaView nanoparticle tracking analyzer (Particle Metrix GmbH) (42). Samples were diluted to 1:1,000 in previously filtered PBS (0.22 μm) for an optimal concentration range for the NTA software (ZetaView Software version 8.02.31). Software parameters were the temperature at 23°C, the sensitivity of 30–85 frames per second (fps), a shutter speed of 55, and a laser pulse duration equal to that of shutter duration. Acquisition parameters were set to a minimum brightness of 20, a maximum size of 200 pixels, and a minimum size of 5 pixels. Polystyrene particles (Microtrac GmbH) with an average size of 100 nm were used to calibrate the instrument before sample readings.

### Transmission electron microscopy

Cells were processed and visualized according to Albergoni et al. (13). EV’s morphology and size were determined by TEM using the negative staining technique (43). Briefly, 5 μL of previously purified EVs was adsorbed for 30 s onto a copper grid (300 mesh) coated with Formvar (polyvinyl resin, Ted Pella, Inc.). Fluid excess was removed with filter paper. Subsequently, the samples were treated with 2.5% uranyl acetate for 30 s and placed in a vacuum desiccator until visualization. EVs were visualized by TEM (FEI Tecnai Spirit) operated at 120 kV. ImageJ software was used to measure the diameter of up to 200 EVs per sample.

### Cell culture of animal cells

Murine alveolar macrophage cell line (ATCC AMJ2-C11—CRL-2456) and human lung adenocarcinoma cell line (ATCC A549—CRM-CCL185) were cultured in Dulbecco’s Modified Eagle’s Medium (DMEM) High Glucose (Gibco, US) containing 10% fetal bovine serum (FBS—Gibco, Brazil) and incubated at 37°C at 5% CO_2_. BMDCs were obtained from C57BL/6 mice as described (44). Female mice (8–12 weeks old) were euthanized via intraperitoneal injection of a mixture of ketamine (400 mg/kg) and xylazine (40 mg/kg). Femurs and tibias from the hind legs were isolated, and the connection points were cut with scissors. Bone marrow from femur bones was obtained and harvested in four petri dishes with 10 mL RPMI 1640, supplemented with 10% FBS (Gibco, Brazil), 1% Pen/Strep (Gibco, US), and 20 ng/mL rGM-CSF (recombinant granulocyte macrophage-colony stimulating factor—Peprotec, US). Cells were incubated for 7 days at 37°C, 5% CO_2_, with an addition of 10 mL of supplemented medium on day 3. On day 7, BMDCs were suspended in RPMI 1640 medium, supplemented with 10% FBS (Gibco, Brazil) and 1% Pen/Strep (Gibco, US). BMDMs were obtained from C57BL/6 mice as described above (45). For BMDMs, bone marrow cells were flushed with RPMI 1640 medium (Sigma Aldrich, US) supplemented with 3% FBS (Gibco, Brazil) and 1% pen/strep (Sigma Aldrich, US). Cells were centrifuged at 200 × *g*, 10 min, 4°C, and then resuspended in 10 mL of RPMI 1640 supplemented with 10% FBS and 20% L929 cell-conditioned medium. Cultivation was carried out for 3 days at 37°C in 5% CO_2_. On the third day, 10 mL of conditioned medium was added and incubated for an additional 7 days. Cells were then centrifuged, the supernatant removed, and the cells suspended in RPMI 1640 supplemented with 10% FBS.

### Proteomics

For S-trap protein digestion, the samples were homogenized with a pestle and probe sonicator in a buffer containing 5% SDS, 5 mM DTT, and 50 mM ammonium bicarbonate (pH = 8), and left on the bench for about 1 h for disulfide bond reduction. Samples were then alkylated with 20 mM iodoacetamide in the dark for 30 min. Afterward, phosphoric acid was added to the sample at a final concentration of 1.2%. Samples were diluted in six volumes of binding buffer (90% methanol and 10 mM ammonium bicarbonate, pH 8.0). After gentle mixing, the protein solution was loaded onto an S-trap filter (Protifi) and spun at 500 *g* for 30 s. The sample was washed twice with binding buffer. Finally, 1 µg of sequencing-grade trypsin (Promega), diluted in 50 mM ammonium bicarbonate, was added to the S-trap filter, and samples were digested at 37°C for 18 h. Peptides were eluted in three steps: (i) 40 µL of 50 mM ammonium bicarbonate, (ii) 40 µL of 0.1% TFA, and (iii) 40 µL of 60% acetonitrile and 0.1% TFA. The peptide solution was pooled, spun at 1,000 *g* for 30 s, and dried in a vacuum centrifuge.

For sample desalting prior to mass spectrometry analysis, the samples were desalted using a 96-well plate filter (Orochem) packed with 1 mg of Oasis HLB C-18 resin (Waters). Briefly, the samples were resuspended in 100 µL of 0.1% TFA and loaded onto the HLB resin, which was previously equilibrated using 100 µL of the same buffer. After washing with 100 µL of 0.1% TFA, the samples were eluted with a buffer containing 70 µL of 60% acetonitrile and 0.1% TFA and then dried in a vacuum centrifuge.

For LC-MS/MS acquisition and analysis, the samples were suspended in 10 µL of 0.1% TFA and loaded onto a Dionex RSLC Ultimate 300 (Thermo Scientific), coupled online with an Orbitrap Fusion Lumos (Thermo Scientific). Chromatographic separation was performed with a two-column system, consisting of a C-18 trap cartridge (300 µm ID, 5 mm length) and a picofrit analytical column (75 µm ID, 25 cm length) packed in-house with reversed-phase Repro-Sil Pur C18-AQ 3 µm resin. Peptides were separated using a 90 min gradient from 4% to 30% buffer B (buffer A: 0.1% formic acid, buffer B: 80% acetonitrile + 0.1% formic acid) at a flow rate of 300 nL/min. The mass spectrometer was set to acquire spectra in a data-dependent acquisition mode. Briefly, the full MS scan was set to 300–1,200 m/z in the Orbitrap with a resolution of 120,000 (at 200 m/z) and an AGC target of 5 × 10e5. MS/MS was performed in the ion trap using the top speed mode (2 s), an AGC target of 1 × 10e4 and an HCD collision energy of 35.

Proteome raw files were searched using Proteome Discoverer software (v2.5, Thermo Scientific) using the SEQUEST search engine. The search for the total proteome included variable modification of N-terminal acetylation and fixed modification of carbamidomethyl cysteine. Trypsin was specified as the digestive enzyme with up to two missed cleavages allowed. Mass tolerance was set to 10 pm for precursor ions and 0.2 Da for product ions. The peptide and protein false discovery rate was set to 1%. Following the search, data were processed as described before (46). Briefly, proteins were log2 transformed, normalized by the average value of each sample, and missing values were imputed using a normal distribution two SDs lower than the mean. Statistical regulation was assessed using a heteroscedastic T-test (if *P*-value < 0.05). Data distribution was assumed to be normal, but this was not formally tested.

### Quantitative analysis of protein EVs and GO evaluations

From the list of proteins in each sample, initially a Venn diagram was constructed to depict the common proteins detected in both growth conditions, or the exclusive proteins in either growth condition. For the enrichment analysis, from the heteroscedastic Student’s *t*-test (*P* ≤ 0.05) described previously, statistically modulated proteins were categorized into two clusters (enriched in BHI vs. enriched in HMM). Initially, GO functional annotations of the statistically modulated proteins in each cluster (biological process, cellular localization, and molecular function) were retrieved from the *E. africanus* proteome (https://www.uniprot.org/). Annotations were further confirmed using the blast2go software ( https://www.blast2go.com/). For the GO enrichment analysis, the number of enriched proteins from each GO category in each cluster was normalized to the total number of proteins detected in the EVs (universe) to calculate the % query. In parallel, the frequency of proteins from the annotated categories in the whole genome was recorded (% universe). The fold enrichment of each GO category was calculated by dividing the (% query)/(% universe), using the Metwarebio platform (https://cloud.metwarebio.com/; GO enrichment analysis), and comparisons were performed by a modified Fisher’s exact test (EASE test; *P* < 0.05).

### β-1,3-Glucan and chitin-related structures

The quantification of β-1,3-glucan and chitin-related structures was performed using an inhibition ELISA with WGA- and dectin-1-Fc (IgG) fusion proteins (47). Two 96-well polystyrene microplates were used: a reaction plate and an inhibition plate. The reaction plate was coated with 50 µL of chitin oligomers or laminarin (10 µg/mL in PBS, Sigma, US) and incubated overnight at 4°C. After washing three times with PBS, wells were blocked with 1% BSA (Sigma, US) for 1 h at 37°C. A second 96-well polystyrene microplate, inhibition plate, was also blocked for 1 h at 37°C with the same blocking buffer and then washed three times with PBS. For quantifications, a standard curve of chitin oligomers (prepared at 1 mg/mL) or laminarin (prepared at 10 mg/mL in PBS) was made with concentrations ranging from 100 µg/mL to 15.25 ng/mL (obtained by serial dilutions 1:2), and 50 µL of each concentration was added to the inhibition plate in duplicate. Still on the inhibition plate, EVs were plated following the same 1:2 dilution as the standard curve. Fifty microliters of a 4 µg/mL solution of WGA-Fc or dectin-1-Fc chimeras was added to all wells of the inhibition plate, respectively, containing chitin oligomers or laminarin, and then incubated for 2 h at 37°C. The contents of the inhibition plate were then transferred to the respective wells in the reaction plate. Excess chimeras for chitin oligomers or laminarin, or EVs, and then plates were left to interact with the chitin and laminarin of the reaction plate, respectively, for 1 h at 37°C. The wells were then washed three times and were incubated with 50 µL of a 1 µg/mL solution of HRP-conjugated anti-mouse IgG (Southern Biotech, US) for 1 h at 37°C. The plates were then washed three times with PBS and were incubated with 50 µL of 3,3′, 5,5′-tetramethylbenzidine (TMB; Thermo Fisher Scientific, US). The reaction was stopped with 12.5 µL of 1 N HCl (Merck, US), and the absorbance was measured in a microplate reader with a 450-nm filter (Biotek ELx808).

### Cell viability assay

The cytotoxicity of EVs was assessed using the colorimetric MTT assay (3-(4, 5-dimethylthiazol-2-yl)-2,5-diphenyltetrazolium bromide; Sigma, US) (48) in AMJ2-C11, A549, BMDCs, and BMDM. Cells were cultivated in RPMI 1640 or DMEM High glucose supplemented with 10% FBS (Gibco, Brazil) and 1% penicillin-streptomycin (Gibco, US). A total of 2 × 10^5^ cells were plated onto 96-well plates in RPMI-FBS and treated with EVs at concentrations of 0.1, 1, and 10 μg/mL, based on protein content. Plates were incubated at 37°C with 5% CO_2_ for 24 h. After incubation, the supernatant was discarded, and 200 µL of the MTT solution was added to the plates. The plates were then incubated for 4 h at 37°C with 5% CO_2_. The blue formazan crystals were solubilized with 200 µL of dimethyl sulfoxide (DMSO; Sigma, US), and the absorbance was quantified using a microplate reader at 540 nm (Biotek ELx808). The percentage of viability was calculated through the following equation: % viability = (OD_540_ treated group)*100/ (OD_540_ control group). All the assays were performed in quadruplicate.

### Expression of CD40 and MHCII by BMDCs stimulated with *E. africanus* EVs

A total of 2 × 10^6^ BMDCs were added to each well of a 12-well cell plate and incubated overnight at 37°C in 5% CO_2_. BMDCs were then treated with EVs from *E. africanus* at 0.1, 1, and 10 µg/mL protein concentrations for 24 h at 37°C. PBS was used as a control condition. After incubation, wells were washed three times with PBS. Supernatants were discarded, and 1 mL of cold PBS was added immediately to the wells, and the plate was placed on ice. The cells were gently detached by pipetting and then centrifuged at 200 × *g* for 5 min. To avoid non-specific binding, the systems were blocked with 10% FBS and 2% normal mouse serum solution in PBS for 1 h on ice. After washing the cells three times with PBS, the cells were incubated for 1 h on ice with 5 µg/mL of fluorescence-labeled antibodies to CD11c-APC (Biolegend, US), CD40-FITC (Biolegend, US), and MHCII-PE (Biolegend, US) following the manufacturer’s instructions. Cells were washed three times with PBS and fluorescence detected by flow cytometry using a BD LSRFortessa cell analyzer. Data were analyzed with FlowJo Software (v. 10.2). Dendritic cells were gated as CD11c^+^.

### Cytokine production by BMDCs and BMDMs treated with *E. africanus* EVs

A total of 2 × 10^5^ BMDCs or BMDMs were added to each well of a 96-well cell plate and incubated overnight at 37°C in 5% CO_2_. BMDCs or BMDMs were then treated with EVs from *E. africanus* at 0.1, 1, and 10 µg/mL protein concentrations for 24 h at 37°C. The concentration of TNF-α, IL-6, and IL-10 produced by BMDCs and BMDMs was determined in the collected supernatants using an ELISA kit, following the manufacturer’s instructions (BD Biosciences, US). Briefly, 96-well Nunc-Immuno polystyrene maxisorp ELISA flat-bottom plates (ThermoFisher Scientific, US) were coated overnight at 4°C with diluted capture antibodies. Plates were then washed three times with washing buffer (PBS with 0.05% of Polysorbate 20—Sigma, US) and blocked with assay diluent for 1 h at room temperature (RT). After blocking, plates were washed three times and incubated for 2 h at RT with distinct concentrations of cytokine standards or collected supernatant. After washing three times, IgG-peroxidase-conjugated antibodies specific for each cytokine were added. Plates were incubated at 37°C for 1 h, washed seven times, and then incubated with substrate solution, TMB (3,3′,5,5′-tetramethylbenzidine, Thermo, US), for 30 min RT in the dark. A stop solution (H_2_SO_4_ 2N—Sigma, US) was added, and plates were read at 450 nm (Biotek ELx808) to determine the absorbance.

### Killing assay

Killing capacities of BMDMs were evaluated following a previously described method (49). A total of 10^6^ cells (in 400 μL RPMI 1640 supplemented with 10% FBS and 1% pen/strep) were added into wells of a 24-well plate and incubated overnight at 37°C in 5% CO_2_. After incubation, the medium was replaced by fresh medium containing either PBS or *E. africanus* EVs (10 µg/mL, based on protein content) and the plates incubated for an additional 24 h at 37°C in 5% CO_2_. Then, *E. africanus* yeasts were added at an MOI of 1:1, and the plate was incubated for 30 min under the same conditions. Non-adherent yeasts were gently removed by washing the wells three times with PBS, and fresh medium was added. The plates were then incubated for 24 h or 96 h at 37°C in 5% CO_2_. At each time point, the host cells were lysed with ice-cold water for 20 min, and 100 µL aliquots were placed on BHI agar. The plates were incubated at 37°C for 15 days, and the number of fungal colonies was then counted.

### *Galleria mellonella* treatment and infection

*G. mellonella* (final instar larval stage) were selected according to weight (0.25–0.30 g). Larvae (20 per group) were inoculated with 10 µL of EV suspensions (100 µg/mL per insect, based on the protein content) using a 30 G insulin syringe into the hemocoel through the last proleg with the EVs of *E. africanus*. The larvae were then placed in sterile Petri dishes and kept in the dark at 37°C for 2 days. Subsequently, larvae were inoculated with 10 μL of *H. capsulatum* G217b suspensions containing 5 × 10^8^ yeasts/mL (5 × 10^6^ yeasts/insect) into the hemocoel through the last proleg using a 30 G insulin syringe, as described (43). Yeasts were washed with PBS, enumerated, and immediately used to infect the larvae. PBS alone was used as a negative control. All larvae were placed in sterile Petri dishes and maintained in the dark at 37°C. Larvae mortality was monitored daily for 15 days. Death was assessed by the lack of movement in response to stimulation. Data were analyzed with GraphPad Prism 7 software. Two independent experiments were performed.

### Statistical analyses

All experiments were performed and repeated in biological triplicates within two or three independent experimental sets. Statistical analyses were performed using a two-way ANOVA with Tukey’s multiple comparisons test, ordinary ANOVA with Dunnett’s multiple comparisons test, and Log-rank (Mantel-Cox) for survival analysis. All analyses were performed using the program GraphPad Prism 7. In all analyses, *P*-values of 0.05 or lower were considered statistically significant.

## Data Availability

The raw proteomics data have been deposited in the EMBL-EBI PRIDE database (PXD062584). The data set containing the proteomic analyses is available at Mendeley Data (https://data.mendeley.com/datasets/f2574hn637/1).
