# Erratum for Honorato et al., “Extracellular vesicles of *Emergomyces africanus* modulate host immune responses and reflect metabolic adaptations to nutrient availability”

**DOI:** 10.1128/iai.00434-26

**Published:** 2026-08-14

**Authors:** Leandro Honorato, Albaniza Liuane Ribeiro do Nascimento Sabino, Jhon Jhamilton Artunduaga Bonilla, Susana Ruiz Mendoza, Julio Kornetz, Flavia C. G. dos Reis, Elaine C. Albergoni, Vinicius Alves, Susana Frases, Allan Jefferson Guimarães, Daniel Zamith-Miranda, Simone Sidoli, Joshua D. Nosanchuk, Marcio L. Rodrigues, Leonardo Nimrichter

## ERRATUM

Volume 94, no. 3, e00632-25, 2026, https://doi.org/10.1128/iai.00632-25. Byline: Elaine C. Albergoni’s name should appear as shown in this Erratum.

